# MicroRNAs MiR-17, MiR-20a, and MiR-106b Act in Concert to Modulate E2F Activity on Cell Cycle Arrest during Neuronal Lineage Differentiation of USSC

**DOI:** 10.1371/journal.pone.0016138

**Published:** 2011-01-20

**Authors:** Hans-Ingo Trompeter, Hassane Abbad, Katharina M. Iwaniuk, Markus Hafner, Neil Renwick, Thomas Tuschl, Jessica Schira, Hans Werner Müller, Peter Wernet

**Affiliations:** 1 University Düsseldorf, Medical Faculty, Institute for Transplantation Diagnostics and Cell Therapeutics, Düsseldorf, Germany; 2 Howard Hughes Medical Institute, Laboratory of RNA Molecular Biology, Rockefeller University, New York, New York, United States of America; 3 Molecular Neurobiology Laboratory, Medical Faculty, Department of Neurology, University Düsseldorf, Düsseldorf, Germany; Universidade Federal do Rio de Janeiro, Brazil

## Abstract

**Background:**

MicroRNAs are short (∼22 nt) non-coding regulatory RNAs that control gene expression at the post-transcriptional level. Here the functional impact of microRNAs on cell cycle arrest during neuronal lineage differentiation of unrestricted somatic stem cells from human cord blood (USSC) was analyzed.

**Methodology/Principal Findings:**

Expression profiling revealed downregulation of microRNAs miR-17, -20a, and -106b in USSC differentiated into neuronal lineage but not in USSC differentiated into osteogenic lineage. Transfection experiments followed by Ki67 immunostainings demonstrated that each of these microRNAs was able to promote proliferation of native USSC and to prevent in part cell cycle arrest during neuronal lineage differentiation of USSC. Bioinformatic target gene predictions followed by experimental target gene validations revealed that miR-17, -20a, and -106b act in a common manner by downregulating an overlapping set of target genes mostly involved in regulation and execution of G_1_/S transition. Pro-proliferative target genes cyclinD1 (CCND1) and E2F1 as well as anti-proliferative targets CDKN1A (p21), PTEN, RB1, RBL1 (p107), RBL2 (p130) were shown as common targets for miR-17, -20a, and -106b. Furthermore, these microRNAs also downregulate WEE1 which is involved in G_2_/M transition. Most strikingly, miR-17, -20a, and -106b were found to promote cell proliferation by increasing the intracellular activity of E2F transcription factors, despite the fact that miR-17, -20a, and -106b directly target the transcripts that encode for this protein family.

**Conclusions/Significance:**

Mir-17, -20a, and -106b downregulate a common set of pro- and anti-proliferative target genes to impact cell cycle progression of USSC and increase intracellular activity of E2F transcription factors to govern G_1_/S transition.

## Introduction

Unrestricted somatic stem cells (USSC) from human cord blood constitute a rare CD45-negative population capable of inducible homogenous *in-vitro* differentiation into all three germinal layers [1], [2]. Additionally, using a cocktail of growth and differentiation factors (XXL-medium), differentiation of USSC into cells of neuronal lineage (XXL-USSC) expressing neurofilament and sodium channel proteins was obtained [3]. Furthermore, XXL-USSC display certain neurotransmitter phenotypes including expression of GABA [1], dopamine and tyrosine hydroxylase (TH), the key enzyme of the dopaminergic pathway [3]. Yet this neuronal lineage differentiation of USSC appears to be limited since patch-clamp analyses failed to detect voltage activated fast inactivating Na^+^ current [1], [3], indicating that XXL-USSC have not yet developed a fully functional neuronal phenotype. Nevertheless, cultured USSC rapidly stop proliferation upon addition of XXL-medium and such cell cycle exit events are inherently connected to neurogenesis [4].

As a series of coordinated events, the cell cycle consists of distinct phases namely S, M, G_1_, and G_2_. Regulation of the cell cycle is performed by a phosphorylation cascade involving cyclin/CDK complexes and three restriction checkpoints, G_1_/S, G_2_/M and metaphase, which sense flaws in critical stages and subsequently stall cycle progression [5], [6]. Transition from G_1_ to S phase is governed by E2F transcription factors [7] under inhibitory influence of hypophosphorylated retinoblastoma proteins (RB1, RBL1, RBL2, [8]). Retinoblastoma proteins are phosphorylated by Cyclin D1/CDK4/6 complexes [9], which in turn are targets for negative regulation from a variety of effectors from the Cip/Kip family [10] as well as from the INK4a/ARF family [11].

MicroRNAs have received emerging attention over the last years as negative regulators of translation. They constitute a subpopulation of small RNAs of on average 22 nucleotides in length and are initially transcribed as primary microRNAs followed by a two step processing into mature microRNAs and incorporation into the RNA-induced silencing complex (RISC) [12], [13], [14], [15], [16]. MicroRNAs downregulate their target-mRNAs by sequence-specific base-pairing with their 3′-untranslated regions (3′-UTRs) [17], [18], [19], [20], [21] and act as key regulatory molecules in various cellular processes like proliferation, differentiation, apoptosis and metabolism [22], [23], [24], [25], [26].

MicroRNAs also appear as important regulators of cell cycle events [27], [28]. In course of molecular G_1_/S transition regulation, complex relationships including direct microRNA-mRNA interactions and activation of microRNA transcription exist between E2F transcription factors [29], [30], [31] and microRNAs of the miR-17-92 cluster, one of the most intensively characterized microRNA families. Including paralogs, this family consists of miR-17, -18, -19a, -19b, -20a, and -92 (located within a region of 1 kb on chromosome 13), of miR-106a, -19b, -363, and -92 (X-chromosomal) and of miR-106b, -93, and -25 (on chromosome 7) [32]. The miR-17-92 cluster regulates mouse stem cell differentiation [33] and has regulatory potential in leukemia stem cells [34], and stemness genes like CDKN1A and CDKN1C as well as PTEN have been proposed as putative targets [35].

Contradictionary findings about miR-17 functions within cell cycle regulation have been described. Pro-proliferative function has been reported in HEK293T cells and in lymphocytes [36], [37] but an anti-proliferative function has been observed in human breast cancer cells [38]. On the other hand, miR-106b, which shares high sequence homology with miR-17 and miR-20a, was shown to promote cell cycle progression by targeting CDKN1A (also termed p21, [39]). CDKN1A is also targeted by microRNAs of the miR-17-92 cluster [40]. Certain other microRNAs including miR-24, -34a-c, -124, -137, -195, -214, -221, -222, and -372 are also involved in cell cycle regulation at G_1_/M transition as well as at G_2_/M transition (reviewed in [27]).

Here we analyzed the impact of the microRNAs miR-17, -20a, and -106b on cell cycle arrest connected to neuronal lineage differentiation of USSC induced by retinoic acid containing neuronal induction medium XXL. These three microRNAs were found specifically downregulated in XXL-USSC but not in USSC differentiated into osteogenic lineage, which show no cell cycle arrest. Target predictions combined with experimental validations demonstrated that these three microRNAs share a set of target genes of pro- and anti-proliferative nature. We further show that miR-17, -20a, and -106b act in a pro-proliferative manner in USSC and are in part capable to prevent XXL-USSC from cell cycle arrest. In addition, reporter analyses demonstrate an increasing effect of these microRNAs on the activity of E2F transcription factors.

## Results

### Different regulation patterns of microRNAs miR-17, miR-20a, and miR-106b during neuronal lineage and osteogenic differentiation of USSC

**Figure 1:**
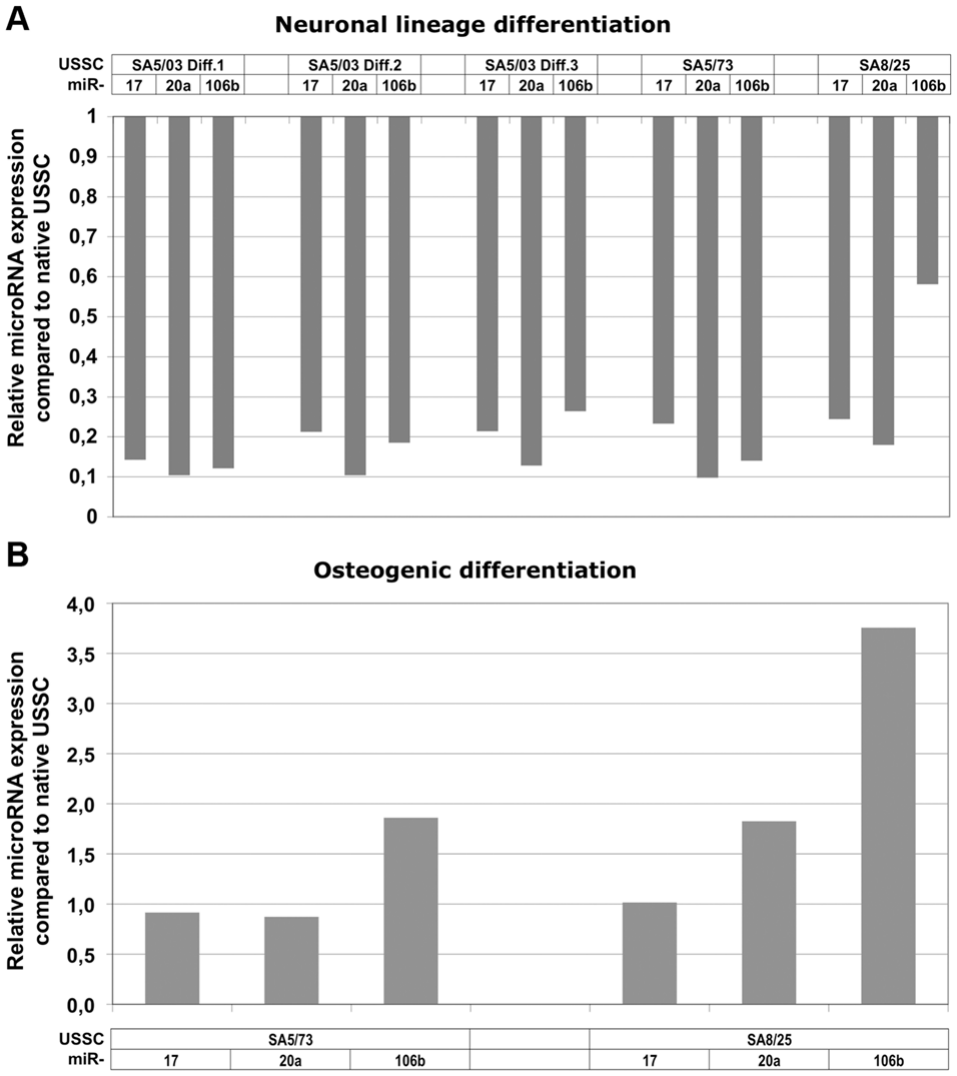
Differential expression of miR-17, miR-20a, and miR-106b in USSC differentiating in neuronal and osteogenic lineages. USSC lines SA5/03, SA5/73, and SA8/25 were differentiated into neuronal lineage for 14 days (SA8/25: 28 days) and USSC lines SA5/73 and SA8/25 were differentiated into osteogenic lineage for 7 days. MicroRNA expression profiles were analyzed from native USSC lines as well as from USSC differentiated into both lineages using the TaqMan microRNA Megaplex array. Fold expression changes (2^−ddCt^-values) are given for microRNAs miR-17, miR-20a, and miR-106b. (A) Downregulation of miR-17, miR-20a, and miR-106b during neuronal lineage differentiation of USSC. Individual results from overall 5 independent neuronal lineage differentiation approaches of USSC lines SA5/03, SA5/73 and SA8/25 are given. MicroRNAs miR-17, miR-20a, and miR-106b were found consistently downregulated in all USSC lines differentiated into cells of neuronal lineage. (B) Expression of miR-17, miR-20a, and miR-106b during independent osteogenic differentiations of USSC lines SA5/73 and SA8/25. In contrast to the neuronal lineage differentiations, expression of miR-17, miR-20a, and miR-106b remained unchanged or was slightly upregulated after 7 days of osteogenic differentiation.

To analyze the impact of microRNAs on cell cycle arrest observed during XXL-mediated neuronal linage differentiation of USSC we compared microRNA expression profiles of XXL-induced USSC with those of USSC induced to osteogenic differentiation. In contrast to neuronal lineage differentiation, DAG-induced osteogenic differentiation of USSC is not coupled with a harsh cell cycle arrest and strong apoptotic events. To analyze and compare microRNA expression, USSC lines SA5/03, SA5/73, and SA8/25 (generated from different donors and serving as biological replicates) were differentiated for 14 days (SA8/25: 14 and 28 days) into neuronal lineage using XXL-medium as described by Greschat and coworkers [3], and USSC lines SA5/73 and SA8/25 were differentiated into osteogenic lineage for 7 days by induction with DAG as described by Kögler and coworkers [1]. MicroRNA expression profiles of native and differentiated USSC of both lineages were determined using the TaqMan microRNA Megaplex qPCR array [41]. As depicted in Fig. 1A, microRNAs miR-17, -20a, and -106b were consistently downregulated up to 10-fold in all XXL-induced USSC lines tested, whereas their expression remained nearly unchanged or was found slightly upregulated in both USSC lines induced to osteogenic lineage (Fig. 1B). Raw qPCR data are shown in Fig. S2. Although the qPCR assay displays high specificity [41], unspecific hybridization of primers or the TaqMan probe between the highly homologous miR-17, -20a, and -106b (Fig. S1) could not be excluded. We thus analysed expression of these microRNAs in native USSC lines SA5/73, SA8/25, SA8/77 and SA4/101 employing a deep sequencing approach and independent expression of miR-17, -20a, and -106b (Fig. S2) in all USSC analysed was detectec here which points to no or only minor unspecific effects of the qPCR assay regarding these microRNAs.

### MiR-17, miR-20a, and miR-106b have common target proteins

Since sequences of miR-17, -20a, and -106b are highly homologous especially with identical seed sequences (Fig. S1), we reasoned that they could act on common target proteins associated with cell cycle regulation. We thus performed extensive target gene predictions using the UNION and the INTERSECTION mode offered by the web-based prediction machine DIANA miRGen [42]. UNION summarizes all predictions from the implemented algorithms, whereas INTERSECTION lists all predictions from algorithms PicTar AND TargetScanS. Lists of predicted proteins were loaded into the DAVID database [43] and analyzed for Gene Ontology (GO) using the search terms *cell cycle* and *prolif*. As seen in Fig. S3, all three microRNAs analyzed share most of the proteins predicted by miRGen's INTERSECTION mode, with only miR-17 lacking a few predicted targets. Predictions using the UNION mode of miRGen add additional proteins, but the large overlap of predicted targets between the three microRNAs remains. These findings suggest a common biological function of miR-17, -20a, and -106b with respect to cell cycle regulation. Interestingly, pro-proliferative as well as anti-proliferative proteins are found among putative target genes.

**Figure 2:**
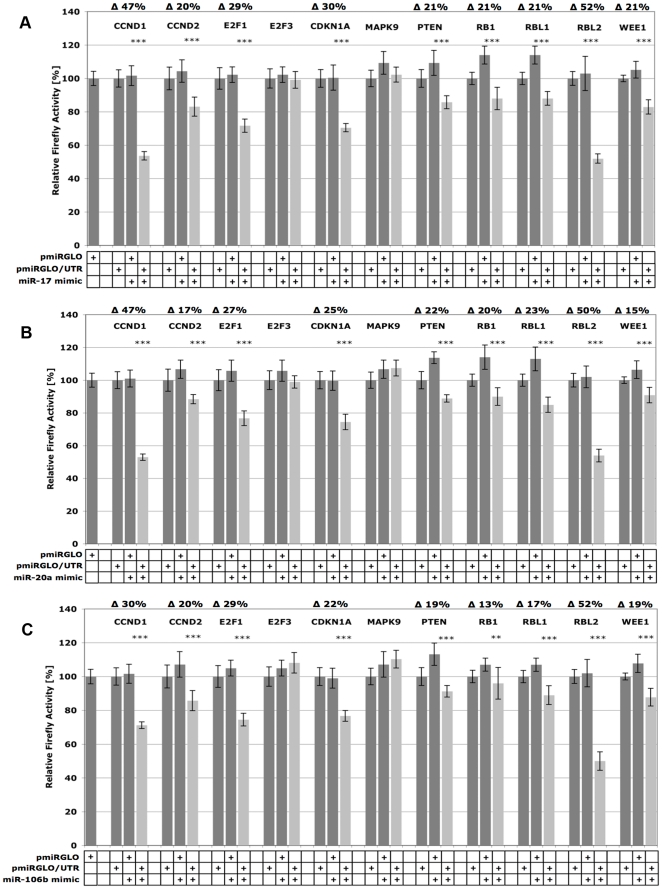
Experimental validation of target gene predictions. Validation of putative cell cycle relevant target genes for microRNAs miR-17 (**A**), miR-20a (**B**), and miR-106b (**C**) in HEK293T-cells. 3′-UTR fragments from putative targets CCND1, CCND2, E2F1, E2F3, CDKN1A, MAPK9, PTEN, RB1, RBL1, RBL2, and WEE1 were cloned at the 3′-end of the *Firefly* ORF in *Firefly/Renilla* dual reporter vector pmirGLO. To test the influence of endogenous microRNAs, pmirGLO and pmirGLO/3′-UTR were each transfected into HEK293T-cells. Normalized *Firefly*-activities were compared to those of pairwise co-transfections of these vectors with the microRNA mimic of interest (miR-17, miR-20a, miR-106b, also including an unspecific mimic negative control) to test for (i) unspecific effects of the given microRNA-mimic on *Firefly/Renilla per se*, (ii) effects of endogenous HEK293T microRNAs (iii) for validation of the particular target prediction. Dark grey columns show normalized *Firefly* activities from pmirGLO/3′-UTR transfections and pmirGLO + mimics co-transfections, light grey columns those from pmirGLO/3′-UTR + mimics co-transfections. Percent reductions of *Firefly* activities from co-transfections of pmirGLO/3′-UTR + mimic compared to pmirGLO + mimic are given as mean values from 2 biological experiments, each consisting of 4 technical replicates. Error bars represent standard deviations and statistical significancies (Student's *t*-test, unpaired, ***: p≤0.001, **: p≤0.01) are given. Effects of the unspecific negative control on pmirGLO and pmirGLO/3′-UTR vectors are shown separately in Fig.S2. Except for E2F3 and MAPK9, all predicted proteins could be validated as statistically significant targets for all three microRNAs tested, although microRNA influence varied from strong (i.e. RBL2) to moderate (i.e. WEE1).

To test, whether the three microRNAs share their biological target proteins beyond the level of bioinformatic prediction, 11 pro- and anti-proliferative proteins from the candidates shown in Fig. S3 covering 33 individual predictions were experimentally validated (Table 1). Namely the pro-proliferative cyclins CCND1 and CCND2, transcription factors E2F1 and E2F3, and the anti-proliferative cyclin D1/CDK4/6-inhibitors CDKN1A and PTEN, retinoblastoma proteins RB1, RBL1, RBL2, and the G_2_/M transition inhibitor WEE1 [44], [45], [46], [47], [48] were analyzed. Some of these proteins (marked with an asterisk in Table 1) have already been validated as targets of miR-17, -20a and/or -106b (CCND1 [38], CDKN1A [39], [40], [49], E2F1 [29], [30], [31], [49], E2F3 [30], PTEN [37], RBL2 [50]) but for reasons of comparability we included them in our experiments. In addition, we analyzed the anti-proliferative MAPK9 not found within the GO-Terms-based list but known as a target for miR-17 [36]. MAPK9 inhibits the cyclin D1/CDK4/6 activator JUN [51]. For experimental target gene validations, full-length 3′-UTRs or fragments of 3′-UTRs (see Table S1) of transcripts of proteins of interest were cloned at the C-terminus of *Firefly* luciferase present in dual luciferase (*Firefly/Renilla*) reporter vector pmirGLO. Pairs of empty pmirGLO and pmirGLO/3′-UTR were cotransfected into HEK293T cells with miR-17, -20a, or -106b microRNA-mimics to test for specific influence of the microRNA on the given 3′-UTR. *Firefly* activities were normalized to effects caused by (i) endogenous HEK293T microRNAs on the 3′UTRs cloned (miR-17, miR-20a, and miR-106b and homologs are highly expressed in HEK293T cells [52]), (ii) unspecific effects of certain microRNA-mimics on *Firefly* and *Renilla* activity *per se*, and (iii) transfection efficiency variations. Percent reductions of normalized *Firefly* activities of co-transfections of each mimic with pmirGLO/3′-UTR compared to pmirGLO are given in Fig. 2A–C. The effects of an unspecific negative control microRNA mimic on all tested 3′-UTRs are summarized in Fig. S4.

**Table 1:** Cell-cycle related proteins predicted as targets for miR-17, miR-20a, and miR-106b and chosen for experimental target gene validation.

hsa-miR-17:	hsa-miR-20a:	hsa-miR-106b:
proproliferative:		
CCND1*	CCND1	CCND1
CCND2	CCND2	CCND2
E2F1*	E2F1*	E2F1*
E2F3	E2F3*	E2F3
antiproliferative:		
CDKN1A*	CDKN1A*	CDKN1A*
MAPK9*	MAPK9	MAPK9
PTEN*	PTEN	PTEN
RB1	RB1	RB1
RBL1	RBL1	RBL1
RBL2*	RBL2	RBL2
WEE1	WEE1	WEE1

Predictions already validated in scientific literature are denoted by an asterisk (*, see main text for details).

Fig. 2A demonstrates that, with the exception of E2F3 and MAPK9, miR-17 significantly influences all target genes tested irrespective of an activating or inhibiting function in cell proliferation. Among the pro-proliferative targets, the strongest effects were seen for CCND1 (appr. 47% reduction in normalized *Firefly* activity) and E2F1 (29% reduction). RBL2 and CDKN1A showed the strongest reductions in activity among the set of anti-proliferative proteins tested (52% and 30% respectively). As seen in Figs. 2B and 2C, miR-20a and miR-106b showed highly similar behavior compared to miR-17 regarding significant *Firefly* activity reductions and relative influences between the analyzed target genes. Overall, these results demonstrate that miR-17, -20a, and -106b not only share a set of common cell cycle associated target genes on the level of bioinformatic predictions but also show highly comparable results in experimental validation of predicted target genes and furthermore affect pro- as well as anti-proliferative target proteins.

### Functional effect of miR-17, miR-20a, and miR-106b on cell cycle arrest in XXL-differentiating USSC

**Figure 3:**
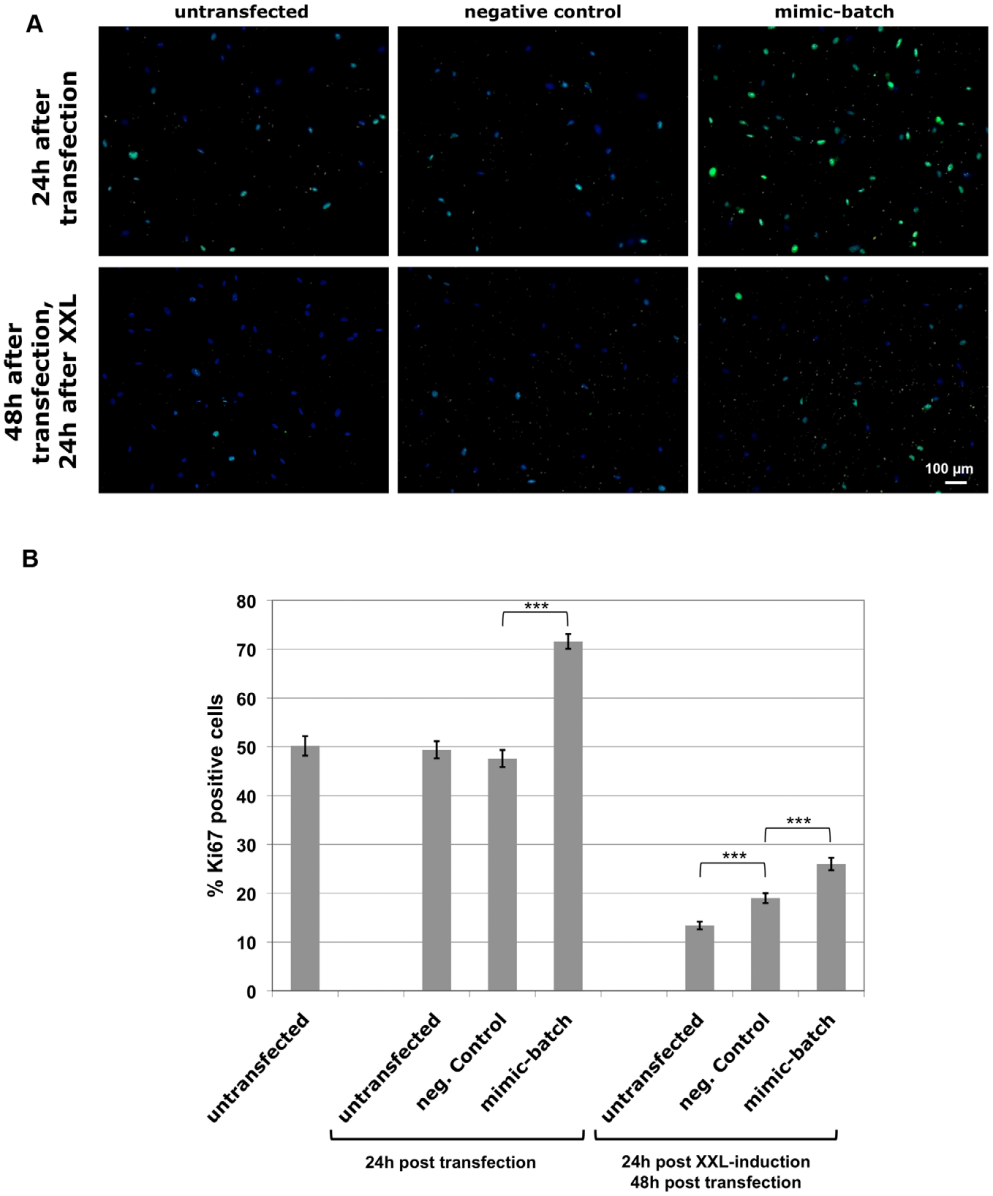
Proliferation-activating effect of microRNA mimics to miR-17, miR-20a, and miR-106b in USSC. (A) USSC line SA5/03 was transfected with a negative control mimic or an equimolar batch of miR-17-, -20a-, and -106b-mimics and induced to neuronal lineage differentiation using XXL–medium 24 h after transfection. Cells were immunostained for proliferation marker Ki67 and DAPI-stained. Ki67 (green)/DAPI stains from time points 24 h and 48 h after transfection are shown from untransfected SA5/03, and from SA5/03 transfected with negative control mimic and the mimic batch, respectively. (B) Increase of Ki67 expression in native USSC as well as in XXL-USSC upon miR-17-, -20a- and -106b-mimics-batch transfection. Combined results from counting of 1500–3000 Ki67/DAPI-stained cells/experimental condition from two biological replicates with 3 technical replicates each are given, together with standard error of the means and statistical significancies (Student's *t*-test, unpaired: ***: p≤0.001).

Since miR-17, -20a (both encoded on the same transcript), and -106b were not only found commonly downregulated in XXL-USSC but also share their target proteins, we chose to analyze the functional impact of these microRNAs on XXL-induced cell cycle arrest. To this end USSC SA5/03 were transfected with 37.5 pmol of an equimolar batch of miR-17-, -20a-, and -106b-mimics, subsequently induced to neuronal lineage by usage of XXL and immunostained for expression of Ki67 antigen before and 24 h after XXL-induction. Ki67 is a nuclear protein expressed by proliferating cells in all stages of the active cell cycle (G1, S, G2, M) but is absent in resting (G0) cells. To collect statistically substantial data, 1500–3000 individual cells per experimental condition from randomly taken photographs of Ki67-stained cells were counted for Ki67 expression. As shown in Fig. 3A, the percentage of Ki67-positive cells increased 24 h after transfection as compared to untransfected cells or those transfected with an unspecific negative control mimic. Twenty-four hours after XXL-induction (and 48 h after transfection), the overall portion of Ki67-positive cells decreased dramatically in untransfected cells and negative-control-transfected cells but was elevated in cells transfected with the miR-17/-20a/-106b-mimic-batch. Fig. 3B shows the results of two independent biological experiments with each three technical replications per experimental condition. Approximately 50% of untransfected USSC were found Ki67-positive before and 24 h after transfection and no significant difference was seen between untransfected and negative-control-transfected cells. Transfection of the mimic batch resulted in a significant increase of Ki67-positive cells to ≥70% of uninduced cells counted. The XXL-induced cell cycle arrest was reflected in a strong drop down of Ki67 expression to approximately 13% Ki67-positive cells 24 h after induction. A strong and significant increase in Ki67 expression was seen upon transfection of the mimics-batch compared to both, negative-control transfected and untransfected cells (Fig. 3B). Taken together, these data demonstrate that a batch of miR-17, -20a, and -106b increases the proliferation rate of USSC and in part prevents them from XXL-induced cell cycle arrest.

**Figure 4:**
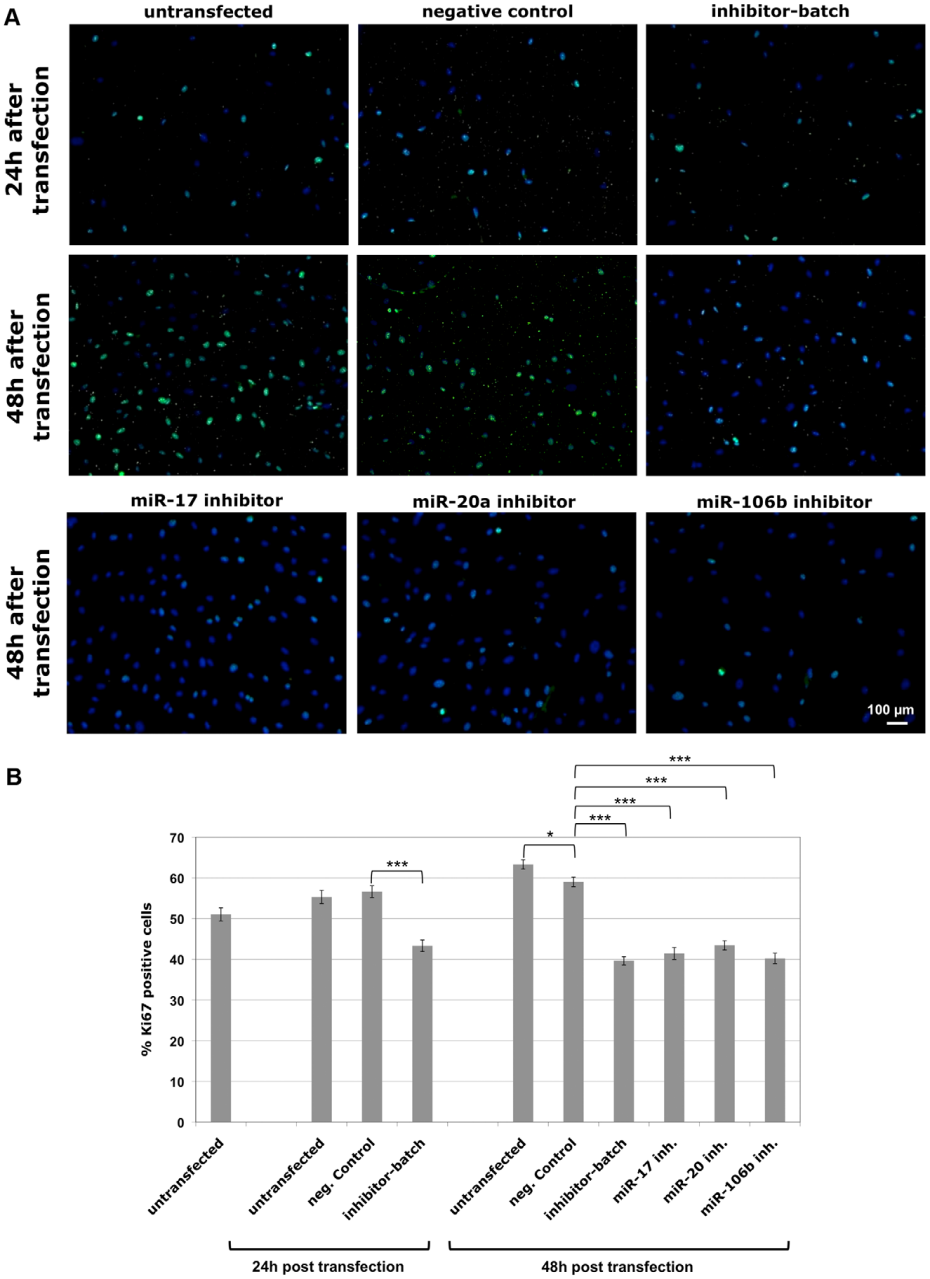
Proliferation-inhibiting effects of microRNA inhibitors to miR-17, miR-20a, and miR-106b in USSC. (A) USSC line SA5/03 was transfected with a negative control inhibitor or an equimolar batch of miR-17-, -20a-, and -106b- inhibitors, as well as with the 3 inhibitors alone. Cells were immunostained for Ki67 expression and DAPI-stained 24 h and 48 h after transfection. Stains from time points 24 h and 48 h after transfection are shown from untransfected SA5/03, and from SA5/03 transfected with negative control mimic and the inhibitor batch, as well as with the miR-17, miR-20a, and miR-106b inhibitors alone (48 h only). (B) Decrease of Ki67 expression in native USSC as well as in XXL-USSC upon miR-17-, -20a- and -106b-inhibitor transfections. Combined results from counting of 1500-3000 Ki67/DAPI-stained cells/experimental condition performed in two biological replicates with 3 technical replicates each are given, together with standard error of the means and statistical significancies (Student's *t*-test, unpaired: *: p<0.05, ***: p≤0.001). Both experimental designs, usage of **microRNA** mimics (Fig. 3A and B) and **microRNA** inhibitors (Fig. 4A and B), demonstrate a proliferation-activating effect of miR-17, -20a, and -106b in USSC.

The inverted experimental design analyzing the impact of microRNA inhibitors instead of microRNA mimics on native USSC showed results fully in line with the aforementioned observations (Fig. 3A and B). Analyzing 1500–3000 randomly photographed cells per experimental condition from each of three independently transfected wells, transfection with 37.5 pmol of an equimolar batch of miR-17-, -20a-, and -106b-inhibitors resulted in a marked decrease of Ki67-positive cells 24 h after transfection as compared to untransfected and negative-control-transfected cells (Fig. 4A and B). A slightly stronger decrease in Ki67-positive cells was observed 48 h after transfection. Replacement of the inhibitor batch by each 37,5 pmol of individual miR-17-, -20a, and -106b-inhibitors and counting two biological replicates with three independently transfected wells each demonstrated, that each of the three microRNA-inhibitors alone was sufficient to fullfill the inhibitory effect on proliferation of USSC with identical efficiency as compared to the combined inhibitor-batch (Fig. 4B).

### A batch of miR-17, miR-20a, and miR-106b increases E2F transcription factor activity in HEK293T cells

**Figure 5:**
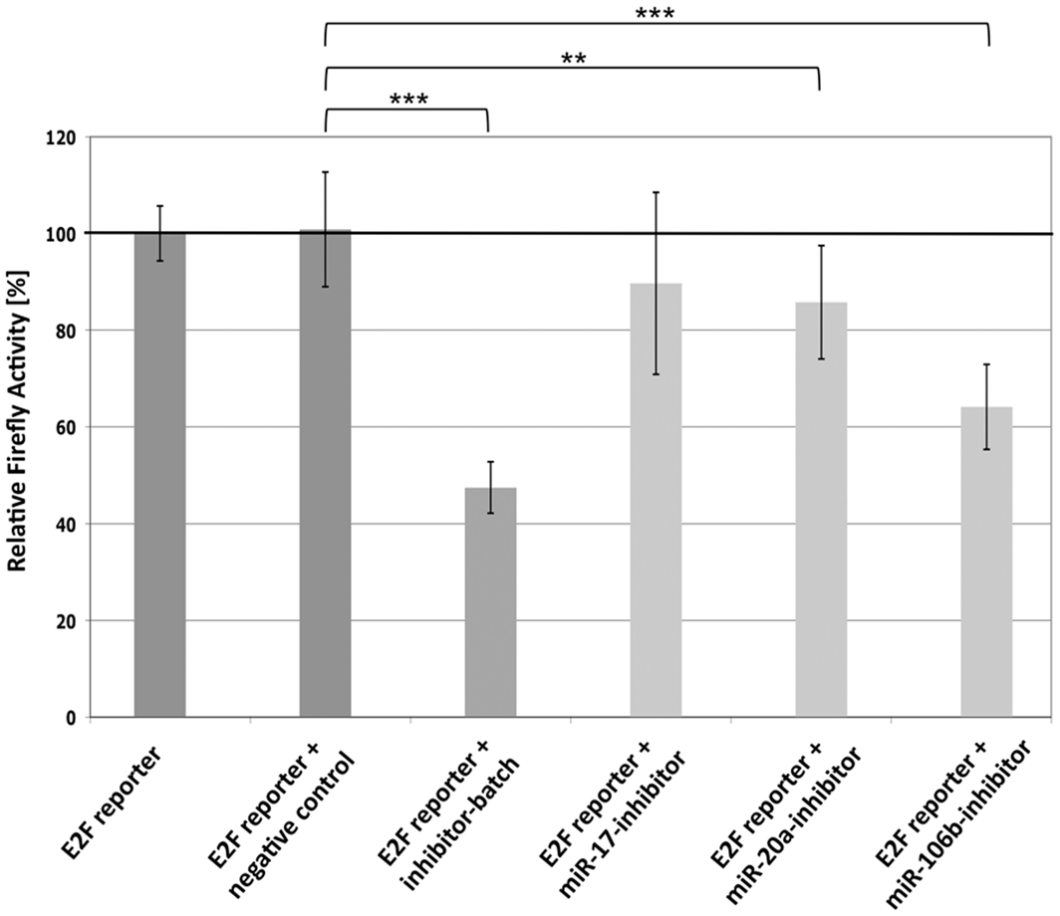
Mir-17, miR-20a, and miR-106b inhibit E2F transcription factor activity. HEK293T-cells were cotransfected with a luciferase reporter vector containing a *Firefly* gene driven by a minimal promoter consisting of a TATA-box preceeded by a repeat of six E2F-responsive elements and pre-mixed with a CMV-promoter driven *Renilla* luciferase reporter vector (Cignal Reporter Assay, SABiosciences) and an equimolar batch of miR-17-, -20a-, and -106b-inhibitors as well as with the inibitors alone and with an unspecific negative control respectively. Mean values of normalized *Firefly* activities from 2 biological experiments, each consisting of 4 technical replicates are shown for each co-transfection. Error bars represent standard deviations and statistical significancies (Student's *t*-test, unpaired: ***: p≤0.001, **: p≤0.01) are given. The inhibitor batch strongly reduced E2F-activity, whereas only miR-20a-, and miR-106b-inhibitor alone gave significant effects on E2F-activity.

Although we have demonstrated a pro-proliferative effect of microRNAs miR-17, -20a, and -106b in USSC, these microRNAs regulate the expression of pro- as well as anti-proliferative target genes in parallel. Especially transcription factor E2F1 was among the most efficiently targeted pro-proliferative proteins identified for the three microRNAs despite transcription factors from the E2F family being major governors of G_1_/S transition. We thus analyzed the impact of miR-17, -20a, and -106b not on the sheer *presence* but on the *activity* of E2F transcription factors using a *Firefly* luciferase reporter driven by a minimal promoter element consisting of a TATA box and a repeat of six E2F-responsive elements. HEK293T cells were co-transfected with this E2F-responsive *Firefly* reporter (plus a *Renilla* reporter vector) together with 3 pmol of an equimolar batch of miR-17-, -20a-, and -106b-inhibitors or with 3 pmol of each of miR-17-, -20a-, or -106b-inhibitors alone. As shown in Fig. 5 the E2F-activity strongly decreased upon addition of the inhibitor-batch compared to the unspecific negative control of the E2F-reporter alone. Interestingly, when testing each 3 pmol of the individual inhibitors alone, only the miR-106b-inhibitor was able to strongly downregulate E2F-activity, whereas the miR-20a-inhibitor showed only moderate effects and the miR-17-inhibitor did not affect E2F activity.

## Discussion

MicroRNAs miR-17, -20a, and -106b were consistently found downregulated in neuronal-specific XXL-USSC but their expression remained nearly unchanged during osteogenic differentiation of USSC. These three microRNAs share high homology within their seed regions, which strongly impacts target mRNA recognition [53], [54], as well as in their 3′-regions (Fig. S1). This implies joint acting of miR-17, -20a, and -106b and consequently, these microRNAs share a large amount of predicted target genes involved in cell cycle events (Fig. S3). Interestingly, these putative targets include both mRNAs of pro-proliferative as well as anti-proliferative proteins, some of which have already been established as targets for miR-17, -20a or -106b (Table 1), using a variety of different reporter gene assays. We chose a group of putative target genes mostly involved in G_1_/S transition for our target validations and, for reasons of comparability, did also include some target genes previously validated in the scientific literature. We did not clone small oligonucleotides spanning only a particular microRNA target site into pmirGLO and also avoided CMV-promoter-driven reporter constructs since in our experience these tend to give false negative results, likely due to prediction errors with regard to the particular microRNA binding site and saturation of the intracellular protein synthesis machinery, respectively.

We were able to validate pro-proliferative proteins like, e.g., CCND1 and E2F1 as well as anti-proliferative proteins like all three retinoblastoma proteins analyzed (RB1, RBL1, and RBL2) and WEE1 as targets for all three microRNAs tested. As could be expected from sequence homologies, miR-17, -20a, and -106b all had comparable effects on the target 3′-UTRs tested. For the first time we identified CCND2, RB1, RBL1, and WEE1 as targets common to all three microRNAs and in addition found new interactions between miR-20a and -106b and CCND1, PTEN, and RBL2. In addition we were able to confirm most of the targets already described for miR-17, -20a, and -106b. Despite their cell cycle relevant target proteins, miR-17, miR-20a, and miR-106b also impact neuronal lineage differentiation of USSC, since certain genes relevant for neuronal differentiation and function like NBEA, EPHA4, NTN4 and NEUROG1 are also affected by these microRNAs [55]. Since cell cycle arrest and neuronal differentiation are linked together [4], this observation suggests a coordinated microRNA impact on both cellular processes.

**Figure 6:**
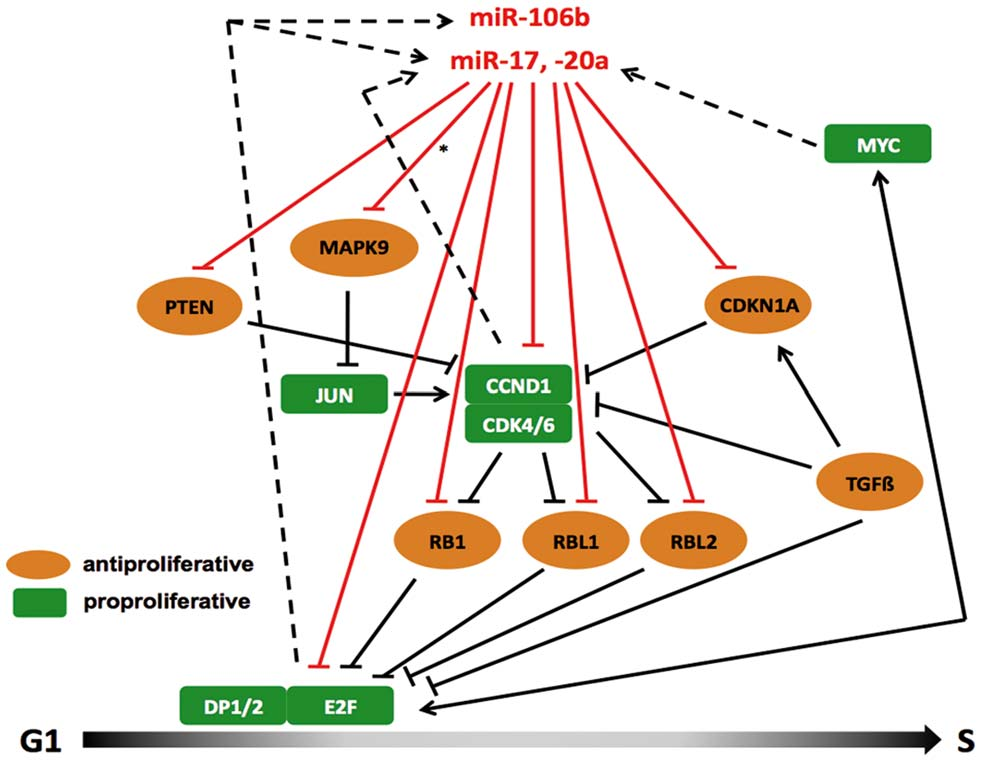
Relationships between pro-proliferative and anti-proliferative proteins involved in G_1_/S transition and miR-17, miR-20a, and miR-106b. Pro-proliferative proteins are shown as green squares, anti-proliferative proteins are given as orange circles. Black lines denote protein-protein interactions, red lines represent microRNA-protein interactions. Dashed black lines show activatory interactions of proteins on microRNA genes. *Predicted interactions of miR-17, -20a, and -106b and MAPK9 could not be validated in our assay, but an interaction between miR-17 and MAPK9 had been shown by Cloonan and coworkers [22].

For a comprehensive overview, Fig. 6 summarizes the relationships between microRNAs miR-17, -20a, -106b and their validated targets in the context of G_1_/S transition. Acting in a joint, putatively additive manner on pro-proliferative as well as on anti-proliferative targets, miR-17, -20a, and -106b are integrated into a complex network of microRNA-protein relations with joint functionality regarding their target genes, protein-protein-interactions and protein-microRNA gene interactions. In general, these three microRNAs target more anti- than pro-proliferative proteins, but their inhibiting effects of pro-proliferative CCND1 and E2F1 were among the strongest effects found within our assays.

By Ki67 expression analysis we demonstrated that these microRNAs are able to override at least in part XXL-mediated cell cycle arrest in neuronal lineage differentiated USSC and clearly act in a proproliferating manner. Interestingly, not only the combined equimolar batch, but also each of these microRNAs alone was able to manipulate USSC proliferation to a comparable extent. These observations strongly point to a pro-proliferative function of each miR-17, -20a, and -106b in USSC and their participation in cell cycle arrest of XXL-USSC, but are of somewhat contradictory nature regarding their aforementioned proproliferating target genes.

As depicted in Fig. 6, G_1_/S transition is executed by transcription factors of the E2F family, namely E2F1-3 [7], [56], which bind to E2F-responsive promoters and are integrated in a complex upstream and downstream regulatory network. Anti-proliferative proteins like CDKN1A and PTEN inhibit kinase activity of CCND1/CDK4/6 complexes and thus prevent phosphorylation of retinoblastoma proteins RB1, RBL1 and RBL2. Hypophosphorylated retinoblastoma proteins in turn associate E2F transcription factors and thereby inhibit cell cycle progression [57], [58]. MiR-17, -20a, and -106b target all mentioned proteins involved in this network, and from the functional interactions described, one “final” goal of this regulatory pathway seems to be the control of E2F activity. In turn, E2F not only activates its own transcription, but is also feed-forward-loop connected (directly or via MYC) to the transcription of miR-17, -20a, and -106b, which has been interpreted as tight cell cycle control mechanism**.** Using a retroviral overexpression approach of the miR-17-92 cluster, Yu and coworkers [38] described an anti-proliferative effect of miR-17 and miR-20a based on their interaction with CCND1-3′-UTR, whereas functional data provided in our study are in line with the earlier report of pro-proliferative effects of miR-17 [36]. These contradictory observations might be explained by a model proposed by Cloonan and coworkers [36], which is based on the relative abundance of pro- and anti-proliferative miR-17-targets. This model can now be extended to miR-20a and miR-106b. It should be noted, however, that microRNA-mediated intracellular regulation not only depends on the abundance of target proteins but also on the relative expression levels of targeting microRNAs and their affinity to 3′-UTRs of target-mRNAs. MiR-17 and miR-20a belong to the medium abundant microRNAs in native USSC and they are found among the most strongly downregulated microRNAs in XXL-USSC, together with miR-106b. Interactions of miR-17, -20a and -106b with CCND1 and RBL2 were among the strongest found in our assays, whereas other target 3′-UTRs showed significantly weaker responses. Since human breast cancers show increased abundance of CCND1 [38], CCND1 might serve as a preferred target in this individual intracellular environment.

In view of these complex relationships, prediction of functional effects of microRNA expression patterns on cell cycle regulation remains difficult, particular since activities of certain proteins involved are regulated by posttranslational modifications or heterodimerization. Therefore, we directly measured the activity of E2F transcription factor as the “executive” protein reponse to inhibition of miR-17, -20a, and -106b and thereby record their downstream “net” effect on E2F activity caused by the interplay of their pro- and anti-proliferative targets. Indeed, activity of a luciferase reporter driven by an E2F-responsive promoter element was strongly reduced upon addition of an equimolar batch of miR-17-, -20a-, and -106b-inhibitors in HEK293T cells. Although single inhibitors were less effective in HEK293T cells than the batch of three inhibitors together (likely due to different cellular expression levels of miR-17, -20a, and -106b in HEK293T cells and different crossreactivities of the single microRNA inhibitors and their highly homologous target-microRNAs (Fig. S1, [59])) this result demonstrates that the inhibitory effects of three microRNAs on anti-proliferative proteins obviously overrides their inhibitory effects on pro-proliferative proteins, especially on E2F1 in HEK293T cells. Due to technical constraints regarding weak plasmid/microRNA inhibitors co-transfection efficiencies in USSC, this experiment could not be performed in USSC, but in view of the results of the Ki67-experiments (Figs. 3 and 4), we assume that USSC follow the same regulatory mechanisms as HEK293T cells.

Pickering and coworkers [31] demonstrated an appoximately 2-fold increase of E2F1 levels upon inhibition of miR-17 and miR-20a in human fibroblasts and a decrease of BrdU–positive cells upon inhibition of miR-17 and miR-20a in serum starved fibroblasts. Our observations are in line with these results and show that microRNAs miR-17, -20a, and -106b can positively modulate E2F activity despite their direct targeting of E2F1. MiR-20a also affects translation of E2F2 and E2F3 [30]. Since E2F1-3 participate in G_1_/S transition and the E2F reporter assay does not distinguish between different E2Fs, it remains unclear, whether miR-17, -20a, and -106b exhibit increasing effects on the activity of E2F1 only or on all E2Fs involved in G_1_/S transition. Nevertheless, the result of the E2F-reporter assay is fully in line with the pro-proliferative effect caused by miR-17-, -20a-, and -106b-mimics and the anti-proliferative effect shown by the miR-17-, -20a-, and -106b-inhibitors in USSC and XXL-USSC, respectively.

All target genes discussed so far are involved in G_1_/S transition, but, in addition, we were also able to identify WEE1 as a target of miR-17, -20a, and -106b. Negative cell cycle regulator WEE1 is a nuclear Ser/Thr kinase which deactivates cyclin B/CDK1 to inhibit the cell cycle at the G_2_/M transition [48]. Although the response of WEE1-3′-UTR in our target gene validation assay was weaker than that of CCND1 or RBL2, this finding further demonstrates, that miR-17, -20a, and -106b can potentially act as pro-proliferative microRNAs in a coordinated manner at different regulatory checkpoints of the cell cycle.

The complex network of cell cycle regulation is further influenced by certain other microRNAs (for an overview see [27]). Among the negative regulators of CCND1/CDK4/6 dimers, members of the Cip/Kip family are regulated by microRNAs. Beside CDKN1A (also termed p21), which is targeted by miR-17, -20a, and -106b ([39], [40], [49], all confirmed in our study), p27 and p57 are downregulated by miR-221 and miR-222 [60]. These microRNAs are also found downregulated in XXL-USSC, as well as miR-137 and miR-214 (Fig. S2), which both target CDK6 [61], [62] In addition to miR-17 [37], miR-20a, and miR-106b (this study), miR-214 also downregulates PTEN [63]. MiR-34a is found upregulated in XXL-USSC (Fig. S2) and is known as a negative regulator of CCND1 and CDK6 [64]. In summary, the differential microRNA expression pattern in USSC and cell-cycle-arrested XXL-USSC clearly point to an anti-proliferative miRNA expression pattern in XXL-USSC. This not only includes the analyzed miR-17, -20a, and -106b, but also additional microRNAs miR-34a, -137, -214, -221, and -222. Although each miR-17, -20a, and -106b alone were sufficient to influence proliferation of USSC and to partly release XXL-USSC from cell-cycle arrest, the additional microRNAs might play a supporting role in keeping XXL-USSC from proliferation.

At first view it is a contradictory finding that inhibition of microRNAs miR-17, -20a, and -106b decreases transcription factor activity of their validated target gene E2F. This demonstrates that a simplistic view of microRNAs as molecular switches needs to be extended to fully understand their molecular function. Rather than being single switches, target gene redundancy of microRNAs as described here for miR-17, -20a, and -106b leads to their integration into complex networks and feedback loops regulating pro- and antiproliferating targets. In such networks, microRNAs in general might fullfill finetuning functions and act as rheostats [19] rather than as molecular switches. Furthermore, target gene redundancy leads to the finding that microRNAs miR-17, -20a, and -106b not only function in cell cycle, but in addition impact neuronal lineage differentiation of USSC together with additional members of the miR-17-92 cluster and paralogs also found downregulated in XXL-USSC [55]. Functional description of microRNAs thus not only requires knowledge of individual microRNA-target gene interactions, but also of abundance of target genes together with their molecular interactions. The data presented here that illustrate the complex interactions within the cell cycle network and help to further understand microRNA function herein.

## Materials and Methods

### Neuronal lineage differentiation of USSC

For expansion, USSC were incubated with DMEM (Lonza, Cologne, FRG) supplemented with 30% heat inactivated foetal bovine serum (Lonza) and penicillin/streptomycin (100 U/ml, Gibco, Invitrogen Company, Karlsruhe, FRG). Neural Differentiation was performed as previously described [3]. Briefly, USSC lines SA5/03, SA5/73, and SA8/25 were seeded on laminin pre-coated glass cover slips and incubated with differentiation medium XXL containing DMEM GlutaMAX™ (Gibco), 15% FBS, U/ml penicillin/streptomycin, 50 ng/ml beta-NGF, 20 ng/ml bFGF (both Tebu), 1 mM dibutyryl-cAMP, 0.5 mM 3-isobutyl-methylxanthine and 10 µM all-trans-retinoic acid (all Sigma-Aldrich, Taufkirchen, FRG) up to 14 days (28 days for SA8/25).

### Osteogenic lineage differentiation of USSC

USSC lines SA5/73 and SA8/25 were induced to osteogenic differentiation as described by [1]. In brief, induction was achieved by addition of DAG (Dexamethason, ascorbic acid, β-glycerolphosphate) and subsequent incubation for 7 days. Osteogenic differentiation was verified by Alizarin-Red staining [1].

### microRNA expression analysis

Preparation of small and large RNA fractions from native USSC and USSC differentiated into neuronal and osteogenic lineages were performed using the Ambion *mir*Vana miRNA Isolation kit (Applied Biosystems, Darmstadt, FRG) according to the manufacturer's instructions, with the sole exception of direct lysis of adherent USSC, thereby omitting cell trypsinisation.

Small RNA fractions were applied to microRNA expression analysis by using the TaqMan microRNA Megaplex array (pool A, Applied Biosystems, Darmstadt, FRG) [41] according to the manufacturer's instructions. Briefly, 10 ng of small fraction sample RNA were reverse transcribed and preamplified for 12 PCR cycles, with a subsequent TaqMan-probe based array-amplification for 40 additional PCR cycles. Raw Ct-values were normalized to U6 RNA data and ddCt as well as 2^−(ddCt)^ data were calculated.

Barcoded small RNA sequencing was used to generate miRNA expression profiles for 4 native USSC lines (SA5/73, SA8/25, SA8/77, and SA4/101); this method is a modification of an established small RNA sequencing protocol which involves sequential ligation of 3′ and 5′ adapters to small RNAs, followed by cDNA library preparation, Solexa sequencing, and small RNA annotation [65].

### Computational target gene predictions

Bioinformatic target gene predictions were performed using web based compilation of several prediction algorithms miRGen (http://www.diana.pcbi.upenn.edu/miRGen.html). MiRGen was used in the UNION mode (collection of all predictions from 6 algorithms) as well as in the INTERSECTION mode (presenting all target genes predicted by algorithms PicTar AND TargetScan), the latter resulting in less but more reliable predictions. The DAVID database [43] (issue 2008, http://david.abcc.ncifcrf.gov/content.jsp?file=fact.html) was used for Gene Ontology and pathway analysis (linked to the KEGG database, http://www.genome.jp/kegg/pathway.html) of predicted target genes. MicroRNA target gene predictions were further completed using the algorithms miRanda ([66], http://www.microrna.org/microrna/home.do), PicTar ([54] http://pictar.mdc-berlin.de/), and TargetScan 5.1 ([17] http://www.targetscan.org/).

### Experimental validation of target gene predictions

PCR-products of full length 3′-UTRs or fragments of 3′-UTRs covering the predicted microRNA binding sites on the target gene mRNA of interest were cloned at the 3′-end of *Firefly* luciferase ORF in dual reporter (*Firefly* and *Renilla* luciferases) vector pmirGLO (GenBank accession FJ376737, Promega, Mannheim, FRG) using restriction enzyme pairs SacI/XbaI or SalI/XhoI. Information on PCR-primers, mRNA templates used and UTR fragment sizes are given in Table S1. To normalize for effects of endogenous microRNAs of HEK293T on the given 3′-UTR, each 100 ng of both pmirGLO vector as well as pmirGLO/3′-UTR were transfected into 5×10^4^ HEK293T cells using 0.5 µl Lipofectamine 2000 (Invitrogen, Karlsruhe, FRG). Pairwise cotransfections of 100 ng empty pmirGLO with the 2.5 pmol microRNA mimic (Dharmacon, Bonn, FRG) of interest and pmirGLO/3′-UTR with the microRNA mimic of interest were performed. *Firefly* and *Renilla* activities were determined 24 h after transfection using Beetlejuice and Renillajuice reagents (PJK, Kleinblittersdorf, FRG). All transfection experiments were performed in at least two independent biological experiments with quadruple transfections each.

### Transfection and immunostaining of USSC

USSC were transfected with microRNA mimics and inhibitors (both Dharmacon, Bonn, FRG) using Lipofectamine 2000 (Invitrogen, Karlsruhe, FRG) according to the manufacturer's instructions. For immunocytochemical analysis of Ki67 expression, cells on coverslips were fixed with 4% formaldehyde (Merck, Darmstadt, FRG) for 10 min, rinsed three times with PBS and incubated with blocking solution including 10% normal goat serum and 0,03% Triton X-100 for 1 h. Cells were incubated with mouse monoclonal α-Ki67 antibody (1∶500, Chemicon, Schwalbach, FRG) and the Alexa 594 conjugated α-mouse secondary antibody (1∶500, Invitrogen). Cell nuclei were labelled with 4,6′-diamidino-2-phenylindoline (DAPI, Roche Diagnostics, Mannheim, FRG). As negative controls and to ensure specificity first antibody was omitted. Photographs were taken from randomly chosen sectors of three independently transfected wells and >1500 cells per experimental condition were counted for Ki67 expression.

### E2F reporter assay

HEK293T cells were cotransfected with 100 ng of E2F-responsive Cignal-vector (SA Biosciences/Qiagen, Hilden, FRG), premixed 40∶1 with a CMV-promoter-driven *Renilla* normalization vector) and 3 pmol of microRNA inhibitors (or 3 pmol of an equimolar batch). *Firefly* and *Renilla* activities were determined 24 h after transfection using Beetlejuice and Renillajuice reagents (PJK, Kleinblittersdorf, FRG).

## Supporting Information

Figure S1 **Sequence Aligning between miR-17, miR-20a, and miR-106b.** The grey box shows the seed regions which are identical in all three microRNAs. Percentages of homology between individual microRNAs are given.
(TIF)

Figure S2 **Raw microRNA expression data.** This Excel file is divided into three sheets “Neuronal diff.”, “Osteogenic diff.”, and “Deep sequencing”. “Neuronal diff.” gives Ct, dCt, ddCT as well as 2^−(ddCt)^-values from neuronal lineage differentiations of USSC lines SA5/03 (3 independent differentiations) SA5/73, and SA8/25 generated by the TaqMan qPCR-assay. Expression data of microRNAs miR-17, miR-20a, and miR-106b and additional cell-cycle-related microRNAs are shown from native USSC as well as from days 14 and 28 (SA8/25) of differentiations. “Osteogenic diff.” gives the TaqMan qPCR-assay data from osteogenic differentiations of USSC SA5/73 and SA8/25 at days 0 (native) and 7. “Deep sequencing” gives deep sequencing expression data of microRNAs miR-17, miR-20a, and miR-106b aquired from native USSC lines SA5/73, SA8/25, SA8/77, and SA4/101. Number of sequence reads as well as expression frequencies are shown.
(XLS)

Figure S3 **List of cell-cycle related genes predicted as targets for miR-17, miR-20a, and miR-106b.** Total predictions from the DIANA prediction tool (http://www.diana.pcbi.upenn.edu/miRGen.html) were filtered using GO terms CELL CYCLE and PROLIF by using the DAVD database (http://david.abcc.ncifcrf.gov/tools.jsp). UNION shows all predictions from all algorithms implemented in the DIANA tool, INTERSECTION shows targets predicted by algorithms PicTar AND TargetScanS within the DIANA webtool.
(XLS)

Figure S4 **Summary of effects of an unspecific negative control on each 3′-UTR used in target validations.** For details refer to Fig. 2. Mean values from 2 biological experiments, each performed in 4 technical replicates are given. Error bars represent standard deviations and statistical significancies (Student's *t*-test, unpaired, *: p≤0.05) are indicated. No significant effect of the unspecific control on any pmirGLO/3′UTR compared to pmirGLO was observed, except for PTEN, where a minor upregulation of normalized *Firefly* activity was observed.
(TIF)

Table S1 **Overview of cloned 3′-UTRs.** A summary of Gene Bank accession numbers, PCR-primers, lengths of 3′-UTRs and cloned fragments thereof used for validations of predicted target genes is given. Restriction enzyme recognition sites are denoted in **bold**.
(XLS)
