# Supplementary figures and images for: MicroRNAs MiR-17, MiR-20a, and MiR-106b Act in Concert to Modulate E2F Activity on Cell Cycle Arrest during Neuronal Lineage Differentiation of USSC
Source: PLoS One. 2011 Jan 20;6(1):e16138. doi: 10.1371/journal.pone.0016138 (PMC3024412; doi:10.1371/journal.pone.0016138)

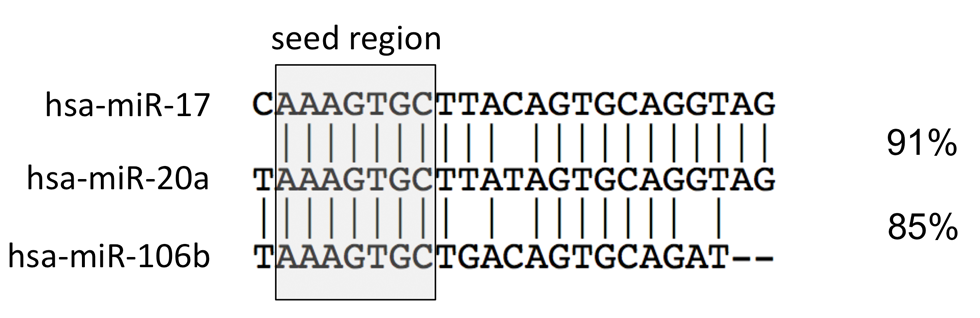

Supplement: Figure S1 — Sequence Aligning between miR-17, miR-20a, and miR-106b. The grey box shows the seed regions which are identical in all three microRNAs. Percentages of homology between individual microRNAs are given. (TIF) [file pone.0016138.s001.tif]

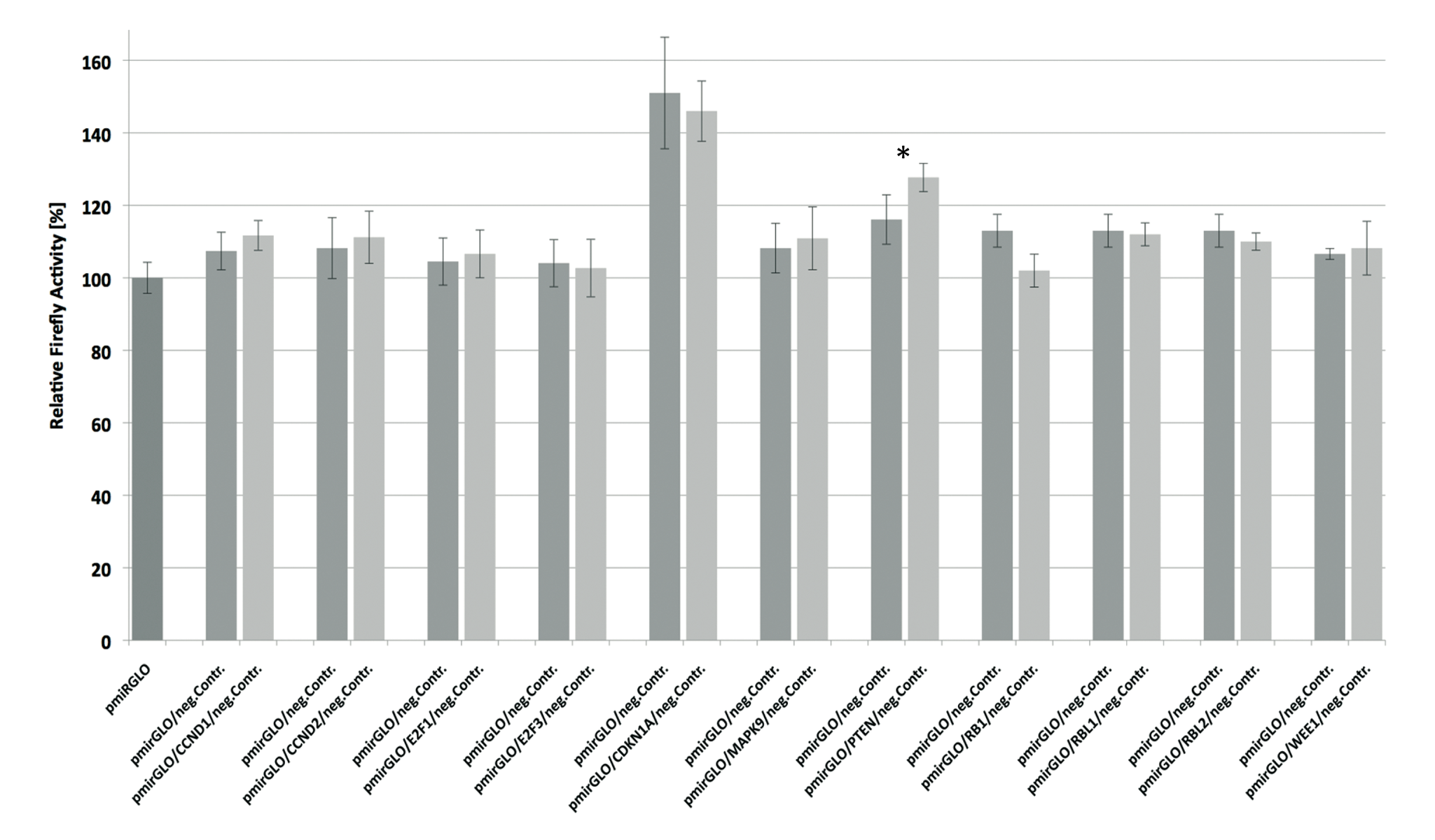

Supplement: Figure S4 — Summary of effects of an unspecific negative control on each 3′-UTR used in target validations. For details refer to Fig. 2. Mean values from 2 biological experiments, each performed in 4 technical replicates are given. Error bars represent standard deviations and statistical significancies (Student's t-test, unpaired, *: p≤0.05) are indicated. No significant effect of the unspecific control on any pmirGLO/3′UTR compared to pmirGLO was observed, except for PTEN, where a minor upregulation of normalized Firefly activity was observed. (TIF) [file pone.0016138.s004.tif]
